# Methods to Characterize Electrospun Scaffold Morphology: A Critical Review

**DOI:** 10.3390/polym14030467

**Published:** 2022-01-24

**Authors:** Alex Lopez Marquez, Iván Emilio Gareis, Fernando José Dias, Christoph Gerhard, María Florencia Lezcano

**Affiliations:** 1 Faculty of Engineering and Health, University of Applied Sciences and Arts, 37085 Gottingen, Germany; alexlopezmarquez@gmail.com (A.L.M.); christoph.gerhard@hawk.de (C.G.); 2 Laboratorio de Cibernética, Departamento de Bioingeniería, Facultad de Ingeniería, Universidad Nacional de Entre Ríos, Oro Verde 3100, Argentina; igareis@ingenieria.uner.edu.ar; 3 Research Centre for Dental Sciences CICO, Department of Integral Adults Dentistry, Dental School, Universidad de La Frontera, Temuco 4811230, Chile; fernando.dias@ufrontera.cl

**Keywords:** scaffold characterization, electrospinning, morphology, porosity, nanofibers

## Abstract

Electrospun scaffolds can imitate the hierarchical structures present in the extracellular matrix, representing one of the main concerns of modern tissue engineering. They are characterized in order to evaluate their capability to support cells or to provide guidelines for reproducibility. The issues with widely used methods for morphological characterization are discussed in order to provide insight into a desirable methodology for electrospun scaffold characterization. Reported methods include imaging and physical measurements. Characterization methods harbor inherent limitations and benefits, and these are discussed and presented in a comprehensive selection matrix to provide researchers with the adequate tools and insights required to characterize their electrospun scaffolds. It is shown that imaging methods present the most benefits, with drawbacks being limited to required costs and expertise. By making use of more appropriate characterization, researchers will avoid measurements that do not represent their scaffolds and perhaps might discover that they can extract more characteristics from their scaffold at no further cost.

## 1. Introduction

Scaffolds are engineered structures designed to imitate the extracellular matrix in order to allow and promote cell growth [1], and creating such structures is the purview of nanotechnology [2]. Electrospun scaffolds are an important focus of regenerative tissue engineering due to their extremely thin fibers with large surface areas, superior mechanical properties, and ease of processing [3]. In essence, the most typical setup for electrospinning consists of a hollow needle tip out of which a polymer solution is ejected. The solution accumulates at the tip. By applying a sufficiently large electric field, a jet is pulled out of the liquid solution, solidifying on its way to the collector plate as the solvent evaporates [4]. The collector plate is often rotated to control fiber alignment and achieve different scaffold morphologies [4,5].

Electrospun fibers have diverse fields of application, for example, filtration and protective material, electrical and optical applications, biomedical applications, sensors, nanofiber reinforced composites, and water purification [3,6,7]. Within the biomedical field, electrospun fibers are applied to tissue engineering [8,9], drug release [9,10,11], wound dressing [12], and monitoring of blood glucose levels [13].

Studies to design, fabricate and characterize fibrous scaffolds have been manifold. The characterization of morphology is essential to the main purpose of such scaffolds: to aid in cell proliferation. The optimal morphology for cell proliferation has been somewhat studied [9,14,15,16,17,18,19,20,21,22,23,24,25,26,27,28]; however, characterization methods that might not be suited for the undertaken characterization are often chosen. Therefore, there is a need to establish a new consensus regarding the means of characterizing the morphology of electrospun scaffolds.

**Figure 1:**
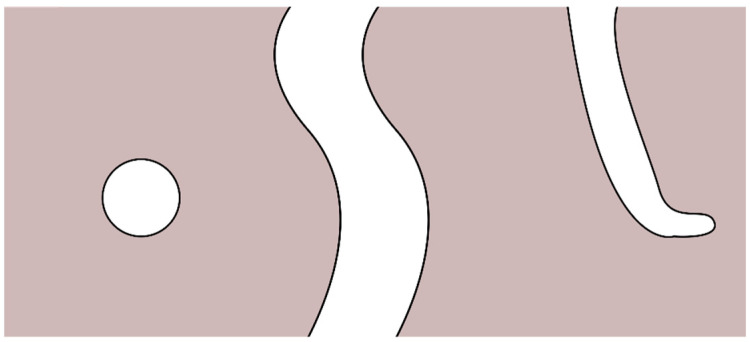
(**left**) Closed pore. (**middle**) Through pore. (**right**) Blind pore. Illustration created with Inkscape.

The morphological characteristics that can be measured in electrospun scaffolds are numerous and complex. Methods traditionally used to measure some of them in other kinds of structures might not apply. In this context, the detection of pores is of essential interest. There are three kinds of pores: blind, through, and closed pores (Figure 1). Methods for characterization are sometimes only capable of accounting for certain kinds of pores. The amount of blind and closed pores in electrospun scaffolds is negligible [19]. Pore size measurement as it is done in the cement industry, where the materials have closed (or isolated) and blind pores [29], does not have the same constrictions as it does for electrospun scaffolds. Moreover, pores are categorized by diameter: pores smaller than 2 nm are micropores, pores between 2 and 50 nm are mesopores, and pores larger than 50 nm are macropores [30]. Micropores influence cell attachment [31] but may not be accounted for by many methods. In addition to pore sizes, other relevant morphological characteristics of electrospun scaffolds include:

- porosity, i.e., the percentage of void space in the scaffold
- pore shape
- pore throat size, i.e., the diameter of the largest opening of a pore
- fiber diameter
- interconnectivity, i.e., the degree to which pores are connected to their neighboring pores
- tortuosity, i.e., the relation between the preferential fluid flow path inside the scaffold and the curvature of its porous structure
- surface area; and
- other less often reported characteristics such as fiber surface topography or fiber alignment (isotropy) [32,33,34].

Apart from cell attachment, pores have a significant impact on the diffusion behavior of scaffold materials as expressed by the effective diffusion coefficient *D_eff_*, given by Equation (1).

(1) $$D_{eff}=\frac{\varepsilon_{t}\cdot \delta \cdot D}{\tau }.$$

Here, *ε_t_* is the number of through pores with sufficient inner diameter for transport of diffusing media, *δ* is constrictivity and *τ* is tortuosity. The accurate and entire characterization of pores is thus an important, but challenging task.

Although reviews exist [35,36,37,38] reporting some methods to measure morphological characteristics, they either do not focus on electrospun scaffolds or do not provide sufficient insight into the relevance of individual characteristics, in addition to most of them not including some of the newer characterization methods available. Furthermore, they do not provide strategical alternatives to somewhat inaccessible methods such as nano-CT.

This review intends to clarify the suitability of current methods to characterize each morphological aspect of electrospun scaffolds, while making the full characterization of scaffolds more accessible with a diverse range of equipment. This should allow authors of future research to choose appropriate methods for characterization, and to find alternatives if they lack access to certain equipment.

## 2. Results

A total of 92 full-text articles were assessed in this review, all in the English language. Table 1 summarizes the reported characterization methods found in the literature and are described in more detail in the following sections.

### 2.1. Physical Methods

#### 2.1.1. Gas Pycnometry

Gas (commonly helium) pycnometry is a method to measure the volume of solids, based on Boyle’s law, also known as Boyle–Mariotte’s law, and is shown in Equation (2).

(2) $$p\propto \frac{1}{V}∴pV=k$$

**Figure 2:**
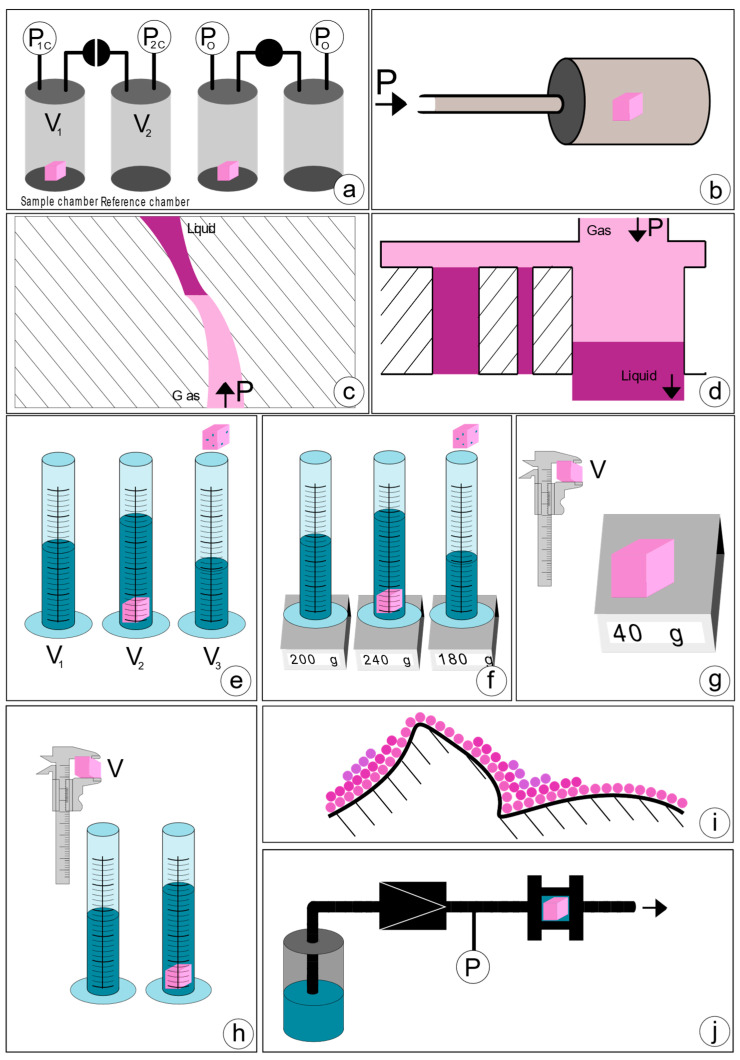
Schematic overview of physical methods for scaffold characterization: (**a**) Gas pycnometry. (**b**) Mercury or liquid intrusion. (**c**) Liquid extrusion. (**d**) Capillary flow. (**e**) Liquid displacement. (**f**) Liquid pycnometry. (**g**) Apparent density method. (**h**) Apparent volume method. (**i**) Gas adsorption. (**j**) Permeability method. Illustrations created with Inkscape.

It states that the product of pressure *p* and volume *V* is some constant *k*. The most basic setup for this approach requires a reference chamber of a known volume connected by a valve to a sample chamber, and a manometer associated to each chamber. First, the pressure of both chambers is measured. Then, the valve connecting the chambers is opened, allowing the pressure to reach an equilibrium. This setup is shown in Figure 2a, where p1C is the initial pressure of the chamber containing the sample, V1 is the gas volume in the chamber, V2 is the volume of the reference chamber, p2C the initial pressure of the reference chamber, and pO is the equilibrium pressure reached after the valve is opened. The volume of the sample can be calculated applying Boyle’s law.

Gas pycnometry allows quantitative assessment of scaffold porosity. The approach to convert the volume to porosity is to measure the apparent volume of a cube that the scaffold has been cut into, using a caliper. The measured pycnometer volume is then inverted and divided by the apparent volume, the result being porosity [39], as shown in Equation (3).

(3) $$porosity=\frac{V_{Apparent}-V_{Pycnometer}}{V_{Apparent}}.$$

Inter- and intra-fiber porosity can be established using He-pycnometry, the results of which are exceptionally accurate to the extent that they account for much of the void space that is unusable for cell infiltration [40]. However, it is often designated as the gold standard for porosity measurement of solids. It does not contain issues with surface hydrophobicity, chemical reactivity and does not damage the structure [41]. Closed pores cannot be characterized by gas pycnometry, but there are virtually no closed pores in an electrospun scaffold. The method has been used multiple times along mercury intrusion porosimetry [42,43], SEM [44], or gravimetry [45] to comprehensively characterize pores. It has also been seldom used to characterize only the density of an electrospun scaffold [46].

#### 2.1.2. Mercury Intrusion Porosimetry

Mercury intrusion porosimetry (MIP) is a method in which mercury is pressured into a porous solid. A basic setup is depicted in Figure 2b. As a non-wetting liquid, mercury does not intrude microscopic pores by capillary action. The required external pressure *p* is directly related to the surface tension γ and the diameter of the pore *D*, as well as the contact angle of mercury *θ*—commonly approximated at 140°, according to Washburn’s equation [47], adapted for the method performed in a vacuum, as shown in Equation (4).

(4) $$p_{Hg}D=-4\gamma cos\theta ,$$

Many morphological properties can be obtained from this method [34], including pore volume, pore size distribution, pore tortuosity and pore/throat ratios. This is performed by not only measuring the intrusion curves, but also the extrusion, providing information about the shape. The size of pores that can be characterized are within the 30 nm–0.2 mm range [48].

It was found that for some micro-scale scaffolds, mercury porosimetry could not provide results due to the potential deformation of the scaffold, caused by the required high pressures [49]. Correction algorithms were developed to improve measurements in structures with pore sizes <10 µm [50]. This was used to characterize scaffolds successfully [51]. MIP has commonly been used to determine porosity and pore size distribution [52], as well as only pore size [53].

Although the results of mercury porosimetry are applicable only to a cylindrical shaped pore, it is assumed that an effective cylindrical pore diameter is being measured when the pores have other shapes [54]. Inkbottle shaped pores (a type of blind pore) might make this assumption an issue [55].

#### 2.1.3. Liquid Intrusion Porosimetry

Liquid intrusion porosimetry is equivalent to MIP, with the exception that other non-wetting liquids such as oil or water are used. Due to the lower viscosity of the liquids applied here and the pressures needed, smaller pores may be measured, as small as 1 nm [48].

#### 2.1.4. Liquid Extrusion Porosimetry

Liquid extrusion porosimetry (LEP) is a method in which a liquid is pushed out of a porous structure by gas. The liquid/gas interface can be seen in Figure 2c. The relationship between the differential pressure Δp, pore diameter *D*, contact angle of the liquid *θ* and surface tension *γ* is described by the Young–Laplace equation [56], Equation (5).

(5) Δp·D=4γcosθ.

Moreover, the technique has been used to derive a distribution function *f_V_* for the pore volume and to calculate the resulting porosity by integrating in all the pore diameter range according to [57]. This is shown in Equation (6).

(6) $$f_{V}=-\frac{dV}{d\;\log D}$$

This method was used to describe pore volume, pore volume distribution, pore shape and specific surface area, but is only applicable to through pores and will not provide proper measurements for blind pores [56]. It can characterize pores in the size range 100 nm–2 mm [48]. This technique assumes for all purposes that pores are cylindrical [54] or spherical [58]. In the examples mentioned above [54,56,58], the fluorocarbon Galwick was used as a liquid agent.

#### 2.1.5. Capillary Flow Porometry (Extrusion Flow Porometry)

Capillary flow porometry (CFP) is similar to LEP, in that gas is used to push liquid out of the structure and the Young–Laplace equation is used. However, the pressure is only increased slowly so as to reach the “bubble point”, at which the largest pores are freed from liquid (see Figure 2d). It is then further gradually increased until the structure is completely dry. The resulting pressure/flow rate curves can be interpreted to provide information on pore throat diameter, pore shape and surface area, but it only measures through pores [56]. However, other researchers found that it was only capable of assessing pore throat size [59]. It can characterize pores of sizes 13 nm–0.5 mm [48].

Moreover, it was reported that deformations of nonwovens produced by the low pressures used in CFP are not significant and not cumulative [60]. The results of CFP, combined with other methods such as LEP, show that pore throat size, pore size and pore diameter increase with increasing fiber width [54]. CFP has been used for providing accurate results in measurements of porosity [61]. It was applied to characterize average pore sizes or their distributions on many occasions [62,63,64,65,66]. It presents issues when characterizing inkbottle shaped (also known as bottleneck) pores [67], however, these are rarely found in electrospun scaffolds.

#### 2.1.6. Liquid Displacement Method

Liquid displacement is a method often used to characterize scaffold porosity [68,69,70]. In the liquid displacement method, the scaffold is added to a known volume of liquid $V_{1}$, commonly ethanol, and is often assisted by various techniques to ensure that all pores are completely filled with liquid. The resulting volume $V_{2}$ is measured, and the impregnated scaffold is removed from the container. The amount of liquid lost to the impregnation of the scaffold $V_{3}$ is recorded, and this volume is equivalent to the void volume. From these measurements, porosity can be calculated according to Equation (7) [71].

(7) $$porosity=\frac{V_{3}}{V_{3}+V_{2}-V_{1}}.$$

This method was also used with hexane as a displacement liquid due to ethanol potentially shrinking the silk scaffold [72]. However, ethanol was previously used on silk scaffolds and the authors did not report shrinkage [73]. Some research indicates that ethanol affects silk structures [74]. The method is schematically depicted in Figure 2e.

#### 2.1.7. Liquid Pycnometry (Archimedes’ Principle)

Liquid pycnometry follows the same steps as the liquid displacement method, with the exception that instead of measuring volumes, weights are measured. The whole procedure takes place in a pycnometer filled with a liquid. First, the weight of the liquid $w_{1}$ and the dry weight of the scaffold $w_{s}$ are measured, then the scaffold is inserted into the liquid, and vacuum or other methods are used to ensure the impregnation of the scaffold. Once this has been achieved, more liquid is added to compensate for the liquid initially displaced by the scaffold until the pycnometer is full again. The weight of liquid and scaffold together $w_{2}$ is recorded, and then that of leftover liquid when the impregnated scaffold has been extracted $w_{3}$. Figure 2f shows a slightly different method which is also reported in the literature, the difference being that not only a pycnometer, but any container can be used. Independent of the liquid density, the porosity can be readily calculated [75,76,77,78,79,80] using Equation (8).

(8) $$porosity=\frac{w_{2}-w_{3}-w_{s}}{w_{1}-w_{3}}.$$

#### 2.1.8. Apparent Density Method (Gravimetry)

In this method, the scaffold is cut into a cube, then its apparent volume *V* is determined with a caliper. The scaffold’s mass *m* is subsequently measured (see Figure 2g), and the resulting apparent density $\rho_{apparent}$ is calculated and compared with the density of the polymer $\rho_{polymer}$, providing the porosity value [81,82,83], according to Equation (9).

(9) $$porosity=1-\frac{\rho_{apparent}}{\rho_{polymer}}$$

This method can be successfully used to determine porosity if the density of the polymer is known, however, identifying the density of an electrospun polymer can be challenging [54] and was reported to be different from that of the bulk material [84]. Moreover, the soft, fine polymers are prone to deformation during caliper measurement. This issue was not tackled in reported studies, instead only the bulk density of the polymer was used [81,82,83,85]. In some studies, accurate results were produced [49].

#### 2.1.9. Apparent Volume Method

The apparent volume method is almost the same as the liquid displacement method, except that the scaffold is cut into a cube and its volume $V_{1}$ is determined by caliper measurements (Figure 2h). The scaffold is then introduced into a known volume of liquid $V_{2}$, after which vacuum pumping or other methods to facilitate impregnation are performed. The final volume $V_{3}$ is recorded, allowing for a slightly different technique to measure porosity [81], calculated by Equation (10).

(10) $$porosity=\frac{V_{1}+V_{2}-V_{3}}{V_{1}}.$$

#### 2.1.10. Gas Adsorption (BET, BJH)

The Brunauer–Emmett–Teller (BET) Theory [86] lays the foundations for a method in which a gas, commonly N_2_, adsorbs to the surface of a measured solid and provides a quantitative assessment of its specific surface area. The data acquired by BET measurements have the form of isotherm curves—with standards described in IUPAC [87] providing information on each adsorption layer. These adsorption layers are schematically shown in Figure 2i, where it is possible to see how a single layer of atoms or molecules adheres to the surface, followed by subsequent layers as described by the so-called Frank–van der Merwe growth [88,89]. Each of these layers has a respective adsorption enthalpy. 

The resolution of this method is extremely high, since it can characterize the surface area of pores as small as 0.5 nm (but only as large as 2 µm) [48]. The method is sometimes used to determine specific surface area [90,91,92], only porosity [93], and sometimes to characterize porosity, mean pore size, and pore size distribution [94,95,96,97,98]. For the purpose of determining pore size and distribution, the Barrett–Joyner–Halenda (BJH) method [99] is used. BET measurement, on the other hand, is known to be associated with great uncertainty [100]. 

#### 2.1.11. Permeability Method

In the permeability method, a liquid is pressed through a solid sample as seen in Figure 2j. The permeability method uses measured permeability by Darcy’s Law [101]. The relationship is described in Equation (11), where *τ* is the permeability, *Q* the volume per time unit, *η* the viscosity, *h* the length of the fluid column, *F* the cross-sectional area perpendicular to the flow, *p* the applied pressure, and *t* represents time:

(11) $$\tau =\frac{Q\eta h}{Ftp}.$$

This approach is used to acquire fiber diameter and pore size [101]. When used on electrospun scaffolds, this method produced accurate results for the fiber diameter, but not for pore size [102].

### 2.2. Imaging Methods

#### 2.2.1. Scanning Electron Microscopy (SEM)

**Figure 3:**
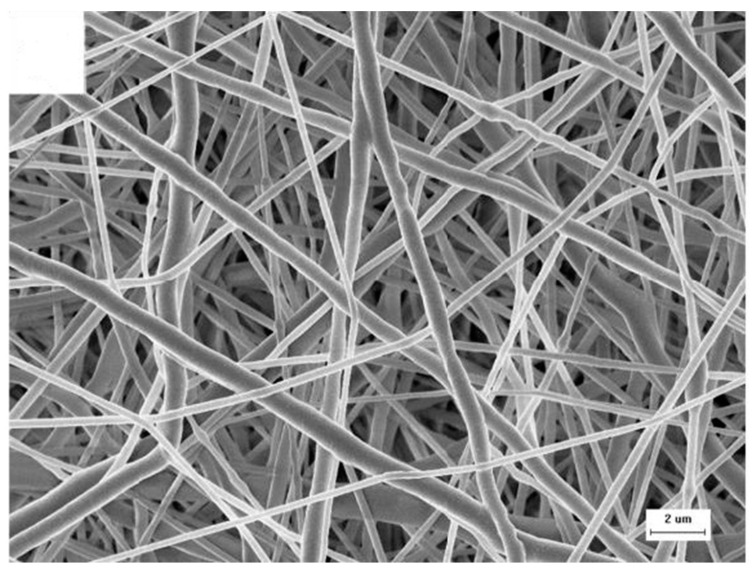
SEM image of electrospun PCL scaffold. Reused from [98] with kind permission from Elsevier.

Scanning electron microscopy is a method in which the surface of a sample is examined, providing information on its morphology. Commonly, to achieve this, an electron beam is directed at the sample, which then excites the atoms on the surface, causing secondary electrons to be emitted. These are then detected, and an image can be constructed. Although there are multiple types of SEM, the most common involves placing the sample in a high vacuum [103]. Samples are often nano sputtered with gold, platinum, or other conductive materials to render the surface conductive and to avoid charging effects. Further preparation steps are often taken in order to ensure the sample is dry and can withstand the low pressure [104]. An advantage of SEM over physical methods is that it can qualitatively assess cell growth on surface layers [105]. The highest attainable resolution of SEM is approximately 1 nm [106]. An example of a typical SEM image of an electrospun scaffold can be seen in Figure 3.

For 3D scaffolds fabricated by stacking electrospun mats, a method was described that used image processing software on the SEM images to determine porosity accounting for the different layers [107]. This method was later used to determine porosity [108], however, the average pore diameter, fiber diameter, and interconnection of the pores were measured using image processing software, meaning those properties of the surface structure were extrapolated to the 3D composition of the scaffold. Another method to produce 3D scaffolds by alternating microfiber with nanofiber mats was also reported. The SEM-measured characteristics included porosity, fiber diameter, and pore size [49]. 

SEM has also been used to characterize scaffold porosity by converting images to binary, whereby fibers are black and pores white or vice versa [109,110]. In some cases, the fiber diameter was also quantified using image processing software.

There are many instances where SEM has been used to measure fiber diameter only, as well as to qualitatively assess morphology [40,54,85]. Occasionally, it is also used to determine mean pore sizes [76,102,111].

One extremely different approach consisted of using a focused ion beam (FIB) to remove surface layer coating and progressively take 2D SEM images of the subjacent layers, allowing for subsequent 3D tomography [112]. It was concluded that the 2D imaging was insufficient in providing insight into the true morphology of the scaffold, however, the 3D method caused results with much noise and was not compared with other 3D characterization methods.

SEM was used to characterize fiber diameter/alignment, pore diameter, and porosity by measuring apparent density from the SEM images and comparing it to the bulk density of the electrospun polymer [113]. A similar method was used later [114], with the exception that porosity was calculated by the physical apparent density method.

Concerns were raised that the electron beam may damage nanometer-scale fibers [115], in addition to the fact that SEM is only capable of characterizing surface properties [106], which might not extend uniformly throughout the scaffold, or even be representative of the interior of an otherwise uniform scaffold. It was reported that the vacuum of the microscope could cause shrinkage of certain samples [116].

#### 2.2.2. Transmission Electron Microscopy (TEM)

Transmission Electron Microscopy (TEM) is a microscopy technique that is equivalent to light microscopy in many aspects, except it uses electrons instead of photons, i.e., light, thus allowing for a much higher resolution due to the easily achievable small de Broglie wavelengths, in addition to presenting minor differences in functionality [117]. However, the optimal thickness for studied samples is in the low nm range [118], rendering it unsuitable for many scaffolds, and only having the potential to examine individual fibers, however with an excellent resolution of down to 0.2 nm [106]. It has been used on individual fibers, especially for fibers containing nanomaterials [119].

#### 2.2.3. Atomic Force Microscopy (AFM)

**Figure 4:**
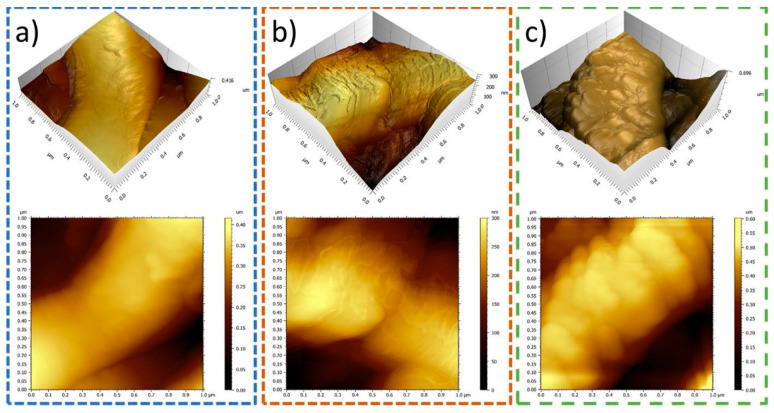
(**a**–**c**) AFM Images of PCL electrospun fiber topography. Reused from [98] with kind permission from Elsevier.

Atomic Force Microscopy (AFM) is a type of microscopy that employs a cantilever probe to physically touch the surfaces, when operated in contact mode. The Coulomb forces exerted on the cantilever by the surface of the sample can then be characterized by piezoelectric components or laser-detector setups. This allows for topographical measurements with resolutions of under 1 nm [120]. When in contact mode, however, the stiffness of the cantilever is superior to that of the bonds present in many polymers. One advantage of AFM is that it allows for the characterization of not only morphological, but of mechanical and even physical–chemical properties. Figure 4 shows a typical AFM micrograph of an electrospun scaffold fiber. In it, 3D renderings of the fiber surface are shown.

AFM was previously used to characterize 2D and 3D surface topography. One important aspect to be noted is that the images show a noticeable variation in fiber diameter [121]. It has also been used to assess the nanostructure of the fibers themselves [122].

Moreover, AFM can be operated in non-contact mode, which might prove advantageous since the nanofibers may be sensitive to the forces exerted by the cantilever. This method was reported for electrospun scaffolds [123]. Another possible operation mode is intermittent contact, or “tapping” mode. Often, it is combined with phase imaging, whereby the shift between the phase of the cantilever and the phase of the response oscillation is recorded. The use of phase imaging has also been reported in the literature, and has provided excellent images of the nanostructure on the fibers as can be seen in [124]. 

As of 2021, AFM is the tool with the highest resolution [106] capable of providing insight into the surface structure of the fibers themselves.

#### 2.2.4. Micro-Computed Tomography (Micro-CT)

Micro-computed tomography is a technique in which X-rays are used to create a 3D image of the structure of a sample. The pixel has a side size in the µm range. This allows for the characterization of µm-scale structures. It can be used in combination with phase-contrast techniques in order to increase its resolution [125]. A significant advantage of micro-CT is the potential of its use on in vivo samples [126,127].

One issue with micro-CT is that the use of higher resolutions leads to smaller scale measurements, which inevitably causes issues when extrapolating the information to the whole structure, especially if a degree of anisotropy is given [128]. The optimal use of micro-CT in tissue engineering, including pixel size, the use of percolation theory and other significant factors has been reported [129]. In this study, however, the criterion for defining the relevance of resolution is the measurement of cells. Considering that the texture of the fibers themselves bear an influence on cell attachment [31], it can be argued that the resolution is insufficient for full morphological characterization.

It was often found that for electrospun nanofibers, the resolution of micro-CT, down to 1–3 µm, was insufficient [54]. In this study, a further difficulty was reported, which was the assessment of a threshold for image processing to identify fibers and pores.

#### 2.2.5. Nano-Computed Tomography (Nano-CT)

**Figure 5:**
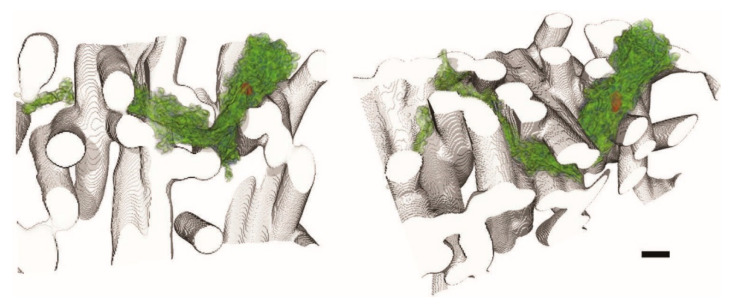
Nano-CT 3D renderings of cells growing on electrospun PLGA scaffold. The cell nucleus is shown in red. The black scale bar is 3 µm. Reused from [132] in accordance with the Creative Commons CCBY License.

Nano-CT uses the same technology as micro-CT, with the pixel sizes being in the nm range [125]. It is commonly used in combination with phase-contrast technology. Resolutions better than 50 nm were already being reported in 2007 [130], making it a tool capable of characterizing many features of nanoscale scaffolds [131], however, many research institutions lack access to nano-CT equipment. In Figure 5, a nano-CT image of cells growing on electrospun scaffolds is shown.

Zernike phase contrast nano-CT was effectively used to fully characterize the morphology of electrospun scaffolds [40], and it has even been used to characterize cell growth on such scaffolds successfully [132,133,134]. The effect of how essential thresholding is to images created by nano-CT can be seen in [135], although only fiber alignment was qualitatively assessed from this image.

#### 2.2.6. Confocal Laser Scanning Microscopy (CLSM)

**Figure 6:**
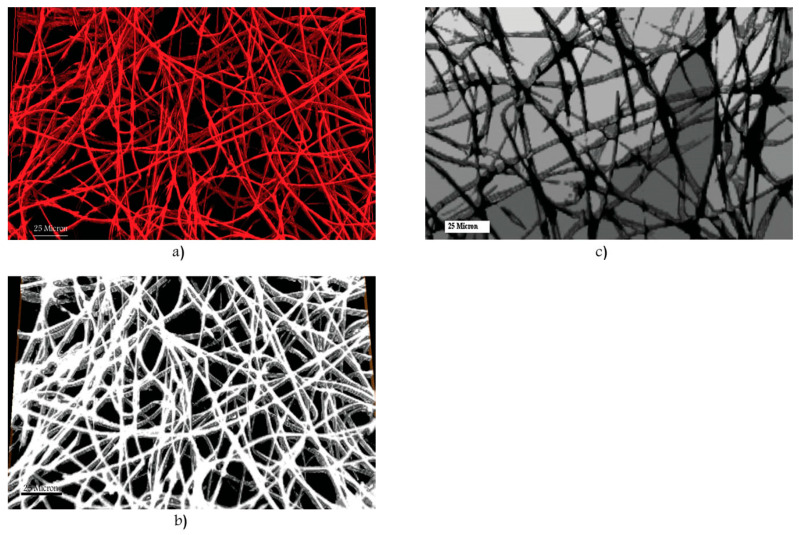
(**a**) Typical 3D CLSM micrograph (**b**) reconstruction from image analysis (**c**) negative image of only pore structure. Images of an electrospun PCL scaffold. Reused from [137] with kind permission from John Wiley and Sons.

Confocal laser scanning microscopy (CLSM) is a technique that allows for spatial filtering and increased resolution of light microscopy through the suppression of light not transmitted directly from the focused part of the sample. It can be combined with fluorescence microscopy and is therefore another powerful tool to image cell growth. Due to the ability of CLSM of obtaining 2D images at different depths, a 3D image can be reconstructed [136]. The resolution of CLSM is about 10 nm on the XY-plane and about 800 nm on the *Z*-axis. It can penetrate samples up to 0.3 mm [106]. Figure 6 depicts typical 3D CLSM micrographs.

The use of CLSM to characterize porous scaffolds was first reported in 2004 [59], but it was used without treating the polymer so as to make it fluorescent. The resulting images were diffuse and unclear, although the authors believed they characterized the scaffold structure better than cryo-SEM. However, due to the diffuse nature of the image it was difficult to extract pore sizes.

In 2013, a novel method of incorporating Quantum Dots—fluorescent nanoparticles—into the polymer solution, along with image post-processing, was introduced [61]. The results mostly coincided with the experimentally and theoretically predicted ones, with fiber volume, total pore volume, porosity, interconnectivity, structure thickness, and average pore throat diameter being measured.

### 2.3. Table of Results

**Figure 7:**
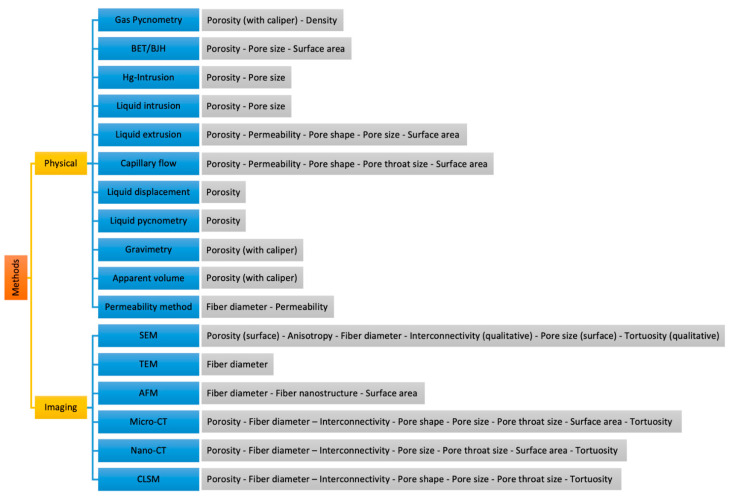
Characterization methods and parameters they can assess.

Table 2 shows the capability of methods reported to assess individual morphological characteristics of scaffolds, according to the information reported thus far. For methods that assessed any other characteristic than porosity, a resolution was reported. The only exception was the permeability method, for which no such data were reported. Figure 7 visualizes the results as summarized in Table 2.

## 3. Discussion

Existing methods for electrospun scaffold characterization were sought from the literature and summarized in Table 1. For each method, reported measured morphological characteristics were found; limitations expressed by the authors or by other existing works were highlighted. This allowed for the completion of Table 2, in which the capability of methods to characterize each feature was evaluated. However, Table 2 is not without its caveats, imposed by the limitations of the methods themselves, in addition to definition issues. These will be explained in detail in this discussion. TEM as a characterization method will not be discussed, since according to the literature, it cannot characterize a multilayer structure. Another characterization method that will not be discussed is liquid intrusion porosimetry, since some studies claim to use liquid intrusion [49,85], but they simply bathed the scaffolds in ethanol, which then “intruded” the structure, allowing for a density comparison method. In this review, this method is classified as “Liquid pycnometry”. Although the use of liquid intrusion porosimetry is theoretically possible, and perhaps even advantageous for networks that compress under the high stress caused by MIP [138], it was not observed in the literature.

Firstly, it is important to consider the purpose of electrospun scaffolds, since the relevance of each characteristic, and therefore the relevance of each method, is dependent on that very purpose. Scaffolds are meant to imitate the extracellular matrix so that they can provide an appropriate cell growth environment [1]. Electrospun scaffolds imitate the extracellular matrix well due to their nanoscale size fibers, since the extracellular matrix contains fiber diameters ranging from 50–500 nm [139], indicating that methods that can characterize this size range are preferable to establish how well a particular scaffold imitates the extracellular matrix. The purpose of measuring scaffolds is therefore twofold: cell growth must be predicted for an existing scaffold, and the scaffold’s architecture must be characterized to correlate cell growth and scaffold architectural parameters. 

Researchers aim to establish which factors are relevant in determining cell growth. Although average pore size is clearly relevant, a pore size distribution might additionally have an effect [140], and although interconnectivity appears important, pore throat size can be an important cell differentiation bottleneck [141]. Permeability measurements might completely forego these measurements by establishing an excellent prediction of cell growth [142], but do not provide useful information on desired scaffold architecture, causing issues with reproducibility. Although other factors such as hydrophobicity are relevant for cell infiltration [143], these can be related to material, fiber, or pore characteristics [144]. Characteristics of the scaffold that result from morphological properties, such as permeability, were not a main concern in this review. Permeability characterization methods for scaffolds were already reviewed elsewhere [142].

As seen in Table 2, almost all methods reported allow for the characterization of porosity. However, the results obtained by different methods may vary wildly, due to the inability of certain methods to account for micropores and their contribution to overall porosity. All methods that use a caliper to determine the apparent volume of a scaffold cube are subject to the issue of scaffold compression by the caliper, since electrospun polymer scaffolds are easily deformed or ruptured, as well as having to be physically sectioned.

Studies attempting to relate cell growth to porosity use a method to determine porosity themselves, creating a possible bias as to what porosity is optimal. However, it seems that increasing porosity is always beneficial to cell growth, unless it leads to scaffold degradation faster than the cell ingrowth rate [22]. This issue might become more significant when comparing the difference in pore size reported by different results, since optimal pore sizes are within a certain range [145]. Although many papers report a pore size distribution, it seems that many physical methods can only characterize pore sizes within a particular range, as can be seen in Table 2. Furthermore, whether the effective pore diameter, actual pore diameter or pore throat diameter is being reported is unclear in capillary flow porometry. The results seem to indicate that it is indeed pore throat diameter [48], however, the data is often used as pore diameter, potentially leading to skewed results when trying to establish a correlation with cell growth. Furthermore, this issue exists in the permeability method, as the authors of [102] reported inaccurate results for pore size. The authors claimed that the issue was probably that what was measured, the pore throat size. However, this cannot account for the full difference since the results significantly differed by a factor of 52.

Imaging methods mostly forego these issues by being able to characterize pore sizes from 3D models, providing measurements for both pore and pore throat diameter. SEM is a notable exception, as it is only capable of qualitatively portraying pore sizes on the surface layer of the scaffold [106]. The combination of FIB–SEM allows for 3D tomography, but is destructive, and due to the random nature of electrospun scaffolds, suffers from issues of reproducibility. However, the advantages that 3D imaging methods provide is additionally overshadowed by the fact that these methods are entirely dependent on thresholding and resolution to provide appropriate 3D images, indicating that they are always an inexact representation of the scaffold itself. For characteristics such as pore size in nanofiber meshes, micro-CT might therefore not be able to provide accurate measurements. Nano-CT, with its improved resolution, seems to tackle this issue. However, with nanofibers <50 nm it also fails to accurately measure fiber diameter, and cannot portray the topography of fibers themselves, which is relevant to cell adhesion and proliferation [146]. Fiber topography, it appears, can only be measured by AFM.

Other than physical and imaging methods, a method to theoretically extrapolate measurements of electrospun scaffolds from few experimental measurements, in addition to evaluation of the relationship between morphological properties, has been described [147]. It was established by the authors that an increased fiber width increased the mean pore radius, but only such relative assessments of the influence of parameters on each other could be made. The authors used a model for paper fibers, applicable solely to randomly-aligned-fiber scaffolds, and cautioned for its use only as a reference, as well as stating it as a qualitative approach [147]. This method was then later used by others to estimate pore sizes quantitively [85]. The method was further expanded by the original authors, showing the relationships between porosity, pore size and specific surface area were somewhat predictable in circular cross-section fiber networks [148]. Research characterizing properties of fibrous networks as a whole [149,150] and the originally proposed theoretical approach for electrospun scaffolds [147] were used to develop a more extensive theoretical approach [151]. The results of this approach were then validated experimentally [151]. A second validation attempt by the same authors resulted in inaccurate measurements [137].

In general, the literature suggests that theoretical methods are only apt for a qualitative extrapolation of certain morphological characteristics of scaffolds from few measurements. They can provide a referential relationship between characteristics, such as the dependency of porosity on fiber diameter, but they cannot accurately predict the pore size distribution of a scaffold. If further work is done, theoretical methods show much promise at providing all morphological characteristics based on a few reliable measurements. They were however not included in this review, considering they are not a proper candidate for scaffold characterization.

Physical methods can have issues such as pore diameter being measured while pore shape is ignored, possibly overlooking detrimental constrictions for growing cells. Although they have some of the highest resolutions of all methods, it is difficult to know what exactly is being measured, and geometrical differences cause artifacts in almost every single method. Moreover, there are potential artifacts that can be encountered in the physical methods that measure porosity, such as the hydrophobicity of the scaffold disallowing water transport into the structure. This can mostly be overcome by using ethanol as the liquid agent if the scaffold material is not affected by ethanol.

Imaging techniques, in general, can best characterize a scaffold. However, knowledge of percolation theory [35], as well as a good understanding of the issue of thresholding [36] is required. There is much research concerning each individual method, particularly micro-CT [152]. Another issue with imaging techniques is that with increasing resolution, smaller areas of interest are imaged. This leads to issues when extrapolating the acquired results to the whole structure. If the structure has an unequal distribution of mass, the results may be inaccurate [128].

One further point to account for is that biodegradable scaffolds will not hold measured properties for long after implantation. As time passes, porosity, surface area and pore diameter will increase. Effectively, this might render scaffolds with pore sizes too small to host cells viable after some time or have the opposite effect in functioning scaffolds. The increase in surface area is desirable, however, the increase in porosity might make the scaffold mechanically unstable if tissue ingrowth is slower than biodegradation [22].

Among the limitations of this review is the inability for these methods to be quantitatively compared with each other. Although this has been carried out experimentally for some methods, as has been explained, what the methods are measuring might not be the same characteristic. The qualitative comparison provided is sufficient to ascertain which methods are generally more beneficial when attempting to evaluate scaffold morphology, but an in-detail comparison of which method is best to evaluate each characteristic has not been performed. The reason for this is the lack of an appropriate method to quantitatively assess scaffold morphology that could serve as a reference for how effectively the methods perform. Often, SEM is used as a reference to compare other methods, and as established in this review, this might be problematic [106]. Although it is almost certain, due to its resolution, that gas adsorption can best characterize surface area, the lack of quantification of associated uncertainties does not allow for such an appreciation. These issues portray the uncertainty associated with comparing existing methodologies.

In future works, researchers can benefit from more appropriate methodological choices for their characterizations. This should lead to a general improvement in the quality of evidence in this field. It is possible that many of the methods included in this review can be further expanded in order to obtain more characteristics from a single measurement. This is especially true for imaging methods, which can be improved with the progress of technology, including the development of better materials, and more advanced modeling and image processing techniques. CLSM is extremely promising and can be used for very detailed characterizations but is not used as commonly as it could be. In particular, with the addition of fluorescent materials into the scaffold, detailed cell behavior analyses can be performed. For instance, a detailed evaluation of the response of cells to nanotopography.

## 4. Conclusions

Due to the inherent limitations of different methods to produce comparable results, it seems that the use of a minimal number of methods is warranted. Nano-CT, with its steadily improving spatial resolution, can provide all morphological characteristics above its resolution range. In terms of assessing a scaffold’s capability for cell growth, its resolution is quite sufficient to accurately portray pore sizes required for all types of cells. However, it is important that the fibers remain above the resolution limit, in addition to proper thresholding being used so as to avoid incurring errors. Although accessibility to nano-CT is limited, due to it being an expensive technology, it currently proves itself as the gold standard for measuring the morphology of scaffolds.

By combining nano-CT with AFM, the topography of the fibers can be studied, providing more insight into the usability of the scaffold. The drawbacks and limitations of other methods to properly assess scaffold morphology make them an alternative that must be handled with care, except for CLSM, which appears to be a viable alternative to nano-CT, at the cost of including fluorescent matter in the scaffold. CLSM can only penetrate samples as far as 0.3 mm, making it a sufficiently capable tool to analyze 3D scaffold morphology for scaffolds that are isotropic in nature.

Physical methods are mostly constrained to which characteristics can be measured with them, as well as the ranges of pore sizes they can characterize. In conclusion, they are not to be preferred. Capillary flow porometry could potentially be employed to complement nano-CT or CLSM measurements, however, there is no need for this in most scaffolds, when these techniques are used correctly. In sub-50 nm fiber scaffolds, however, CFP might prove a useful complementary tool.

Due to some nano-scaffold fibers being smaller than the resolution of the imaging methods, it is advisable to consider which method to use according to the fiber diameter, which can be measured by SEM. SEM, the most widely used tool to characterize scaffolds, should only be used for qualitative assessments in 3D scaffolds, other than for the characterization of fiber diameter.

Future research can benefit from more appropriate methodological choices. CLSM and nano-CT are promising technologies which, currently remain underused for characterizations, and could provide complete measurements and illustrate cell–scaffold interactions with a high level of detail. 

## Figures and Tables

**Table 1:** Methods for scaffold characterization reported in the literature.

Method Type	Method
Physical	Gas pycnometry
	Gas adsorption (BET, BJH)
	Mercury intrusion porosimetry
	Liquid intrusion porosimetry
	Liquid extrusion porosimetry
	Capillary flow porometry
	Liquid displacement method
	Liquid pycnometry
	Apparent density method
	Apparent volume method
	Permeability method
Imaging	Scanning Electron Microscopy (SEM)
	Transmission Electron Microscopy (TEM)
	Atomic Force Microscopy (AFM)
	Microcomputed Tomography (Micro-CT)
	Nanocomputed Tomography (Nano-CT)
	Confocal Laser Scanning Microscopy (CLSM)

**Table 2:** Parameters that each method introduced can assess and their relevant characteristics. M*: Mixed or inconclusive reports in the literature. N: This method cannot be used for this characteristic. Y: This method can be used for this characteristic.

	Porosity	Pore Sizes	Pore Shape	Surface Area	Pore Throat Size	Fiber Diameter	Interconnectivity	Tortuosity	Other Parameters	Particularities
**Gas Pycnometry**	With caliper	N	N	N	N	N	N	N	Density	
**BET/BJH**	Y	Y	N	Y	N	N	N	N		Resolution: 0.5 nm–2 µm
**Hg-Intrusion**	Y	Y	N	N	M*	N	N	M*		Resolution: 30 nm–0.2 mm Toxic, destructive, might deform nanoscaffolds
**Liquid intrusion**	Y	Y	N	N	M*	N	N	M*		Resolution: 1 nm–20 µm
**Liquid extrusion**	Y	Y	Y	Y	N	N	N	N	Permeability	Resolution: 100 nm–2 mm
**Capillary flow**	Y	M*	Y	Y	Y	N	N	N	Permeability	Resolution: 13 nm–0.5 mm
**Liquid displacement**	Y	N	N	N	N	N	N	N		
**Liquid pycnometry**	Y	N	N	N	N	N	N	N		
**Gravimetry**	With caliper	N	N	N	N	N	N	N		
**Apparent Volume**	With caliper	N	N	N	N	N	N	N		
**Permeability method**	N	M*	N	N	N	Y	N	N	Permeability	
**SEM**	Only Surface	Only surface	N	N	N	Y	Qualitative	Qualitative	Anisotropy	Resolution: 1 nm. Can image cells. Can only assess surface of scaffold. Vacuum might shrink scaffolds.
**TEM**	N	N	N	N	N	Y	N	N		Only usable on single fibers. Resolution: 0.2 nm
**AFM**	N	N	N	Y	N	Y	N	N	Fiber nanostructure	Resolution: <1 nm
**Micro-CT**	Y	Y	Y	Y	Y	Y	Y	Y	All within resolution	Resolution: 1–3 µm. Thresholding difficulty. Can image cells.
**Nano-CT**	Y	Y	Y	Y	Y	Y	Y	Y	All within resolution	Resolution: <50 nm. Thresholding difficulty. Can image cells.
**CLSM**	Y	Y	Y	N	Y	Y	Y	Y	All within resolution	Resolution: 10 nm XY-plane, 800 nm Z-Axis. Scaffold must be made fluorescent, can image cells

## Data Availability

Not applicable.
